# Influenza vaccination of healthcare workers in acute-care hospitals: a case-control study of its effect on hospital-acquired influenza among patients

**DOI:** 10.1186/1471-2334-12-30

**Published:** 2012-02-01

**Authors:** Thomas Bénet, Corinne Régis, Nicolas Voirin, Olivier Robert, Bruno Lina, Silene Cronenberger, Brigitte Comte, Brigitte Coppéré, Philippe Vanhems

**Affiliations:** 1 Hospices Civils de Lyon, Hôpital Edouard Herriot, Service d'Hygiène, Epidémiologie et Prévention, Lyon, France; 2 Université de Lyon, université Lyon 1, CNRS, UMR 5558, Laboratoire de Biométrie et Biologie Evolutive, Equipe Epidémiologie et Santé Publique, Lyon, France; 3 Hospices Civils de Lyon, Hôpital Edouard Herriot, Service de médecine du travail, Lyon, France; 4 Hospices Civils de Lyon, Centre National de référence des virus influenza région Sud, Bron, France; 5 Université de Lyon, université Lyon 1, Département de Virologie, Bron, France; 6 Hospices Civils de Lyon, Hôpital Edouard Herriot, Service de médecine gériatrique, Lyon, France; 7 Hospices Civils de Lyon, Hôpital Edouard Herriot, Service de médecine interne, Lyon, France

## Abstract

**Background:**

In acute-care hospitals, no evidence of a protective effect of healthcare worker (HCW) vaccination on hospital-acquired influenza (HAI) in patients has been documented. Our study objective was to ascertain the effectiveness of influenza vaccination of HCW on HAI among patients.

**Methods:**

A nested case-control investigation was implemented in a prospective surveillance study of influenza-like illness (ILI) in a tertiary acute-care university hospital. Cases were patients with virologically-confirmed influenza occurring ≥ 72 h after admission, and controls were patients with ILI presenting during hospitalisation with negative influenza results after nasal swab testing. Four controls per case, matched per influenza season (2004-05, 2005-06 and 2006-07), were randomly selected. Univariate and multivariate conditional logistic regression models were fitted to assess factors associated with HAI among patients.

**Results:**

In total, among 55 patients analysed, 11 (20%) had laboratory-confirmed HAI. The median HCW vaccination rate in the units was 36%. The median proportion of vaccinated HCW in these units was 11.5% for cases vs. 36.1% for the controls (*P *= 0.11); 2 (20%) cases and 21 (48%) controls were vaccinated against influenza in the current season (*P *= 0.16). The proportion of ≥ 35% vaccinated HCW in short-stay units appeared to protect against HAI among patients (odds ratio = 0.07; 95% confidence interval 0.005-0.98), independently of patient age, influenza season and potential influenza source in the units.

**Conclusions:**

Our observational study indicates a shielding effect of more than 35% of vaccinated HCW on HAI among patients in acute-care units. Investigations, such as controlled clinical trials, are needed to validate the benefits of HCW vaccination on HAI incidence in patients.

## Background

Hospital-acquired influenza (HAI) is associated with significant morbidity and mortality in hospitalised patients [1]. Prevention and control of nosocomial influenza entail multiple measures; vaccination of healthcare workers (HCW) is advocated by the Centers for Disease Control and Prevention to obviate influenza transmission in healthcare settings [2]. Influenza vaccine coverage of HCW remains low despite these recommendations [3], indicating that additional data are needed to establish the benefits of vaccination and promote it among HCW.

No evidence of a protective effect of HCW vaccination on proven HAI in patients has been reported. Indeed, the efficacy of HCW vaccination in preventing influenza among patients in long-term care hospitals was not found in a systematic review by Thomas et al. [4] in 2006. Earlier, 2 randomised clinical trials [5,6] disclosed reductions in overall mortality and influenza-like illness (ILI) among elderly patients after HCW vaccination without a decrease in laboratory-confirmed influenza. However, data are sparse in acute-care settings. Studies in long-term institutions were conducted to assess the effectiveness of flu vaccination in preventing disease spread among elderly populations residing in care facilities [4-6]. These settings differed from those in acute-care hospitals in terms of population, care and contact patterns.

Despite such conclusions, HCW vaccination is expected to actually have a protective influence. Simulation studies have shown that HCW vaccination is effective and that its proportion is linked linearly to the influenza attack rate in nursing homes [7] as well as in acute-care hospitals [8]. Clinical trials are the gold standard for addressing this issue, but their results are not conclusive [4-6]. Clustered, randomised trials are difficult to conduct in acute-care hospitals because of rapid patient turnover.

The objective of our observational investigation was to ascertain the effectiveness of influenza vaccination of HCW on laboratory-confirmed HAI among patients.

## Methods

### Setting

Data were extracted from a hospital-based, prospective surveillance study of ILI detailed elsewhere [9]. Briefly, this prospective surveillance study enrolled all ILI patients hospitalised between October 15 and April 15 in 2004-05, 2005-06 and 2006-07, in Edouard Herriot Hospital (Lyon, France), a tertiary, acute-care university hospital with 1,000 beds. Totally, 36 (84%) of the hospital's 43 adult short-stay units participated on a voluntary basis: 12 with 224 beds in 2004-05, 29 with 493 beds in 2005-06, and 30 with 537 beds in 2006-07. No specific infection transmission control measures were implemented during the study period. Standard procedures were followed throughout the hospital, with droplet precautions in case of ILI. Once a day, research nurses contacted participating units to search for new patients with ILI, defined as rectal or axillary temperature ≥ 37.8 C, in the absence of antipyretics, with cough or sore throat [10]. All incident cases of ILI during hospital stay were included. At ILI diagnosis, the following variables were analysed: date of admission and discharge, underlying diseases, start and end dates of clinical ILI features, potential sources of exposure to influenza within 5 days before ILI or influenza occurrence, outcome, influenza vaccination status and vaccination date. On the day of ILI suspicion, nasal swab testing served to confirm influenza in cases and controls. When incident ILI was detected (in patients or HCW), a research nurse visited the unit twice daily to document case follow-up and to find secondary cases, which were included in the surveillance according to clinical criteria similar to those for other ILIs. Each secondary ILI case was followed as an incident case, and the whole unit was monitored for 10 days after incident case onset to detect secondary cases. A potential influenza source was defined as the presence of at least 1 person with ILI (patient or HCW) in the units considered as being contagious, within 5 days before onset-from 1 day preceding ILI to 4 days thereafter.

A nested case-control investigation was conducted in this prospective surveillance study of hospital-acquired ILI [9]. Cases were patients with virologically-confirmed influenza occurring ≥ 72 h after admission in acute-stay units [1]. Controls were patients with ILI during hospitalisation with negative influenza results after nasal swab testing. Four controls per case, matched per influenza season (2004-05, 2005-06 and 2006-07), were randomly selected among all eligible controls. The exposure of interest was the proportion of HCW vaccinated against influenza in these units, collected prospectively by the occupational health department of the hospital. HCW vaccination was confirmed by occupational health department data. All HCW categories were included: physicians, nursing staff, ancillary and allied health staff. The proportion of vaccinated HCW was dichotomised according to the median of its distribution. Potential confounders were gender, likely sources of influenza in the same ward (other patients or HCW) within 5 days before influenza or another ILI occurrence during hospital stay, individual patient vaccination against influenza, type of ward, and underlying disease. Clinical suspicion of influenza was based on the above definition.

### Virological tests

All cases and controls underwent similar nasal swab testing by the *Centre national de référence des virus influenza région Sud *in Lyon. Specimens were analysed for influenza virus by immuno-capture ELISA, immuno-staining, tissue cell culture, and polymerase chain reaction. Sample suspensions were inoculated into Madin-Darby canine kidney cells, epithelial Hep-2 cells and fibroblastic MRC-5 cells. The number of HCW and vaccinated HCW per participating unit as well as per year was recorded prospectively.

### Statistical analysis

Categorical variables were compared by Fisher's exact test, and continuous variables, by the Mann-Whitney *U*-test. Conditional logistic regression was undertaken to assess factors associated independently with HAI among patients. Covariates with *P *< 0.15 after univariate analysis as well as gender and age were entered into the initial multivariate model, backward step-wise regression analysis was conducted, and the likelihood ratio test compared models until *P *< 0.15. Potential interactions between variables of the final model were tested 2 by 2. All tests were 2-tailed, and *P *< 0.05 was considered significant. The data were analysed by Stata software 8.0 (StataCorp LP 2003, College Station, TX). The protocol design was approved by the hospital's institutional review board. All patients received study information and gave signed, informed consent.

## Results

### Population characteristics

Among 55 patients analysed overall, 11 (20%) had laboratory-confirmed HAI. Median age was 77.3 years, and 41 (75%) were female. Totally, 13 (23%) were hospitalised in a medical unit, 8 (15%) in a surgical unit, 8 (15%) in an obstetrics-gynaecology unit, 26 (47%) in a short-term elderly patient unit, and none in an intensive care unit. Seven (13%) had chronic pulmonary disease, 26 (47%) had chronic cardiac disease, and 2 (4%) were immuno-compromised. One patient died during hospitalisation (unrelated to influenza). Twenty (36%) patients had at least 1 potential patient or HCW source of influenza infection in the same ward within 5 days before ILI or influenza occurrence. The median HCW vaccination rate in the units was 36% (0% to 67%) with a mean of 34%.

### Factors associated with HAI among patients

**Figure 1:**
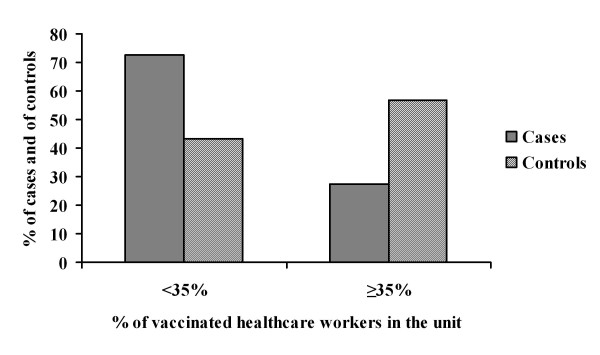
**Repartition of cases and controls according to the proportion of vaccinated healthcare workers in short-stay units of Edouard Herriot Hospital, Lyon (France), in influenza seasons 2004-05, 2005-06 and 2006-07**.

Figure 1 shows the repartition of cases and controls according to HCW vaccination rates in the units. The median proportion of vaccinated HCW in these units was 11.5% for cases vs. 36.1% for the controls (*P *= 0.11). After univariate analysis (Table 1), the presence of at least 1 influenza source in the ward increased the risk of HAI. After multivariate analysis (Table 2), a proportion of vaccinated HCW of more than 35% in the units, compared to a lower proportion, was protective against laboratory-confirmed HAI among patients (odds ratio = 0.07; 95% confidence interval 0.005-0.98), after adjusting for age and the presence of potential influenza sources in the units.

**Table 1:** Characteristics of the controls and patients with laboratory-confirmed, hospital-acquired influenza (HAI) and of the factors associated with HAI in Edouard Herriot Hospital, Lyon (France), in influenza seasons 2004-05, 2005-06 and 2006-07

Characteristics	Patients with confirmed HAI (N = 11)	Controls (N = 44)	*P*	Crude OR^a ^(95% CI)
Gender, female	8 (73)	33 (75)	1.0	0.89 (0.22-3.72)

Age, years				0.99 (0.97-1.02)^b^

Median (range)	66.0 (48.5-85.1)	78.7 (42.3-86.7)	0.6	

Type of ward			1.0	

Medicine	8 (73)	31 (70)		1.0 (Ref.)

Surgery	3 (27)	13 (30)		0.90 (0.21-3.89)

Chronic pulmonary disease	2 (18)	5 (11)	0.6	1.60 (0.31-8.25)

Chronic cardiac disease	5 (45)	21 (48)	1.0	0.92 (0.26-3.28)

Immuno-depression	2 (18)	1 (2)	0.10	8.00 (0.73-88.22)

Potential influenza source in the unit^c^	7 (64)	13 (30)	0.08	4.06 (1.08-15.33)

Individual influenza vaccination^d, e^	2 (20)	21 (48)	0.16	0.34 (0.069-1.70)

Proportion of vaccinated HCW in the unit			0.10	

< 35%	8 (73)	19 (43)		1.0 (Ref.)

≥ 35%	3 (27)	25 (57)		0.16 (0.018-1.32)

Data are reported as numbers (%) under descriptive analysis and as OR (95% CI) under logistic regression analysis. Controls were hospitalised patients with ILI and confirmed exclusion of influenza diagnosis after nasal swabbing; they were matched by influenza season (2004-05, 2005-06 and 2006-07). HAI, hospital-acquired influenza; OR, odds ratio; CI, confidence interval; HCW, healthcare workers.

^a^Under univariate conditional logistic regression

^b^Per 1 year older

^c^Potential sources of exposure to influenza (other patients or HCW) for 5 days before ILI or influenza occurrence in the same ward

^d^In current influenza season

^e^Individual influenza vaccination was not known in 1 patient

**Table 2:** Factors associated independently with hospital-acquired influenza among patients in Edouard Herriot Hospital, Lyon (France), in influenza seasons 2004-05, 2005-06 and 2006-07

Characteristics	Adjusted OR^a ^(95% CI)
Age, per 1 year older	1.03 (0.99-1.07)

Potential influenza source in the unit^b^	5.22 (1.08-25.22)

Proportion of vaccinated HCW in the unit	

< 35%	1.0 (Ref.)

≥ 35%	0.07 (0.005-0.98)

OR, odds ratio; CI, confidence interval; HCW, healthcare workers.

^a^Multivariate conditional logistic regression, adjusted on the other covariates

^b^Potential sources of exposure to influenza (other patients or HCW) for 5 days before ILI or influenza occurrence in the same ward

## Discussion

The study objective was to assess the effectiveness of influenza vaccination of HCW on laboratory-confirmed HAI among patients based on prospectively-collected observational data. Our results indicate that a proportion of vaccinated HCW of more than 35% in short-stay units, in comparison to a lower proportion, was protective against laboratory-confirmed HAI among patients, independently of flu season, patient age, and the presence of a potential influenza source.

Simulations have shown that HCW vaccination is effective against influenza in patients from nursing homes or in acute-care settings, but have provided no evidence of a threshold decrease in the influenza attack rate with increasing HCW vaccination proportion [6,7]. In our investigation, because of its small sample size, we were unable to research a true dose-response relationship (Figure 1). The threshold that we reported was based on the distribution of HCW vaccination in our hospital. A proportion of vaccinated HCW of less than 35% seemed to have no effect on HAI in patients, whereas a proportion of more than 35% could be considered, according to our data, as the minimal vaccination coverage with potential protective impact on hospital-acquired incident cases. This result discloses the difficulties encountered in quantifying the influence of vaccination in clinical trials without taking different HCW vaccination proportions into account. Moreover, 1 simulation study reported an unexpected absence of herd immunity against influenza in nursing homes, implying that the vaccination of every additional HCW protected an additional fraction of patients [7]. Also, while a higher proportion of vaccinated HCW is expected, it has not been demonstrated that the concept of herd immunity, which needs large and closed populations, can be applied in acute-care settings with multiple different contacts.

Our study had some strengths. It was prospective, with case definition based on laboratory-confirmed influenza and standardised definitions regarding the delay to hospital acquisition [1] as well as virological testing of all controls. Also, HAI could be difficult to diagnose. Our study allowed us to analyse the effectiveness of a vaccination strategy, whereas clinical trials with such an objective would have to be very large (i.e., for simulation, 169 departments per arm would be needed) [7]. Simonsen et al. proposed case-control studies with highly-specific endpoints, such as laboratory-confirmed influenza [11]. We attempted to provide more arguments to a question that remains to be answered, namely: does influenza vaccination of HCW have an impact on the occurrence of hospital-acquired, virologically-proven influenza among patients in acute-care hospitals? The topic is of great concern in the context of flu pandemics and even seasonal epidemics in healthcare settings. The experimental design for obtaining a definitive answer might be difficult to implement. Thus, convergent results from observational studies would be helpful for infection control professionals [12].

Our study design also had limitations. The main limitations were the classification bias of exposure and the balance of confounders. Other limitations must be emphasized. First, the number of cases was small because our case definition was restrictive, which constrained our study's power. However, the misclassification bias of cases and controls must have been low because all controls were tested for influenza virus. Second, it is possible that units where a higher proportion of HCW are vaccinated are also units where high standards of respiratory hygiene and infection control practices are respected, and could be important in reducing the risk of nosocomial influenza transmission. However, our investigation was observational, and no additional specific measure was formally implemented because of the study. Finally, exposures of hospitalised patients to visitors were not recorded. They may constitute another non-assessed source of exposure to influenza.

## Conclusions

In summary, our observational study indicates a protective influence of vaccination of more than 35% of HCW on HAI in patients. Other experimentally-designed investigations are needed to demonstrate the effectiveness of HCW vaccination in the control of influenza outbreaks in healthcare settings, and to determine the threshold for vaccinated HCW proportion with more accuracy [13]. Our findings must not be misinterpreted. To date, the HCW vaccination rate of 35% is not optimal to control HAI. Higher vaccination coverage among HCW with strict adherence to recommended infection control measures is warranted, including the screening of visitors and HCW for illnesses suggestive of influenza, the restriction of those with any symptoms from the hospital, the use of neuraminidase inhibitors for the treatment of cases and for the chemoprophylaxis of exposed patients as well as non-vaccinated HCW.

## Competing interests

Bruno Lina received unrelated grants from MedImmune, GSK, Novartis, and Sanofi-Pasteur. All other authors declare that they have no competing interests regarding this study.

## Authors' contributions

TB, CR, NV and PV contributed to study design and data analysis. TB drafted the initial manuscript. OR, BL, SC, BCom and BCop contributed to data analysis and interpretation. All authors read, commented on and approved the final manuscript version.

## Pre-publication history

The pre-publication history for this paper can be accessed here:

http://www.biomedcentral.com/1471-2334/12/30/prepub
